# The Janus-Faced Role of Antioxidants in Cancer Cachexia: New Insights on the Established Concepts

**DOI:** 10.1155/2016/9579868

**Published:** 2016-08-24

**Authors:** Mohamad Assi, Amélie Rébillard

**Affiliations:** EA1274 Laboratory “Movement, Sport and Health Sciences” (M2S), University of Rennes 2, ENS Rennes, 35170 Bruz, France

## Abstract

Chronic inflammation and excessive loss of skeletal muscle usually occur during cancer cachexia, leading to functional impairment and delaying the cure of cancer. The release of cytokines by tumor promotes the formation of reactive oxygen species (ROS), which in turn regulate catabolic pathways involved in muscle atrophy. ROS also exert a dual role within tumor itself, as they can either promote proliferation and vascularization or induce senescence and apoptosis. Accordingly, previous studies that used antioxidants to modulate these ROS-dependent mechanisms, in cancer and cancer cachexia, have obtained contradictory results, hence the need to gather the main findings of these studies and draw global conclusions in order to stimulate more oriented research in this field. Based on the literature reviewed in this paper, it appears that antioxidant supplementation is (1) beneficial in cancer cachectic patients with antioxidant deficiencies, (2) most likely harmful in cancer patients with adequate antioxidant status (i.e., lung, gastrointestinal, head and neck, and esophageal), and (3) not recommended when undergoing radiotherapy. At the moment, measuring the blood levels of antioxidants may help to identify patients with systemic deficiencies. This approach is simple to realize but could not be a gold standard method for cachexia, as it does not necessarily reflect the redox state in other organs, like muscle.

## 1. Introduction 

Approximately, 50% of patients with advanced stage of cancer experience cachexia and more than the third die following the loss of ~75% of skeletal muscle mass [1]. Cachexia is defined as a multifactorial syndrome characterized by a loss of more than 5% of total body weight mainly due to skeletal muscle wasting with or without depletion of adipose tissue [2]. Thus, the management of cancer cachexia is primordial to achieve a successful treatment. Pharmacological agents and single-nutritional interventions proposed to treat cachexia mainly resulted in an increase of fat mass but failed to effectively restore lean body mass [3, 4]. Indeed, muscle wasting is the component of cachexia that has the greatest negative impact on quality of life and anticancer treatment efficiency [2], hence the need to ameliorate our knowledge and understand the underpinning molecular mechanisms involved in cachexia-associated muscle catabolism.

Reactive oxygen species (ROS) are highly reactive, unstable, and short-lived molecules that play a crucial role in both health and disease [5]. Physiological amounts of ROS are produced endogenously (e.g., mitochondrial respiratory chain) and intervene in essential physiological mechanisms including phagocytosis, redox signaling, neurotransmission, proliferation, differentiation, and apoptosis [6–8]. Contrariwise, in pathological conditions, excessive ROS levels could lead to the development of oxidative stress (OS). OS is defined as a “disruption of the redox balance towards an increase in prooxidant over the capacity of antioxidants, leading to a perturbation of redox signaling and control and/or molecular damage” (i.e., lipids, protein, and DNA) [5, 9].

Several pieces of evidence suggest a key role for ROS in the development of muscle atrophy in response to the inflammatory profile related to cancer cachexia [10, 11]. Importantly, ROS also exert a double-faced role in tumor through triggering either growth/progression or death [8]. Accordingly, a number of clinical and preclinical studies of cancer and cancer cachexia have used antioxidants including vitamins E and C, *β*-carotene, *α*-lipoic acid, carbocysteine, and N-acetylcysteine, to antagonize or modulate these ROS-sensitive mechanisms. Unfortunately, the obtained results were not always positives but sometimes without any significant effect or even deleterious [12–19]. Indeed, if the use of antioxidants appears to be complicated in cancer, it could be even more problematical in cancer cachexia given the intricate tissue crosstalk and the disruption of redox balance that takes place in many organs, including skeletal muscle, heart, liver, and blood [17, 20, 21]. In other words, high levels of ROS could be present at different sites, at the same time, and exert distinct roles in an organ-dependent manner. For example, the inhibition of ROS could be beneficial in skeletal muscle to reduce the magnitude of atrophy but deleterious within tumor as this may accelerate proliferation and growth [17, 22]. This multiorgan presence of ROS confers to cachexia an overelaborate nature and, thus, makes the intervention with antioxidants more perplexing.

Additionally, the self-prescription and uncontrolled use of supplements by patients may distort the conclusions regarding benefits or harms of antioxidant supplementation. Epidemiological studies have shown that more than 50% of patients increase their consumption of complements after diagnosis of cancer, without any medical prescription [23]. Antioxidant and nutritional supplements are used by cancer patients as they believe that these compounds feature a powerful anticancer activity [24]. Definitely, an adequate uptake of multivariate/multicolor fruits and vegetable is necessary for a healthy life-style and the world cancer research fund (WCRF) advise cancer patients to obtain antioxidants from food rather than supplements [25], whereas the random consumption of high-doses antioxidant complements is a real threat for cancer patients, as it can alter the efficacy of anticancer therapies and negatively influence tumor growth [26]. The use of antioxidants in cancer and cachexia has always been a polemical issue, hence the need to gather the main existing knowledge in an attempt to answer a number of essential questions and improve our understanding on this topic: how can the undifferentiated use of supplements by cancer patients impact tumor and anticancer treatment? Can some tumor types also benefit from antioxidants? How can we improve the use of such compounds? What is the factor that will provide eligibility for a cancer patient to undergo antioxidant supplementation?

## 2. Multiorgan Presence of Oxidative Stress Markers during Cancer Cachexia: Skeletal Muscle, Blood, Heart, and Liver

*Clinical Studies*. Oxidative damage markers were increased in the skeletal muscle of cachectic patients. Specifically, patients with lung cancer exhibited an increase in the levels of protein carbonyls in* vastus lateralis*, which correlated positively with muscle proteolysis [28]. The interesting study from Buck's team showed that lipid peroxidation adducts, malondialdehyde (MDA), were elevated within skeletal muscle (i.e.,* vastus lateralis*) of patients with colon, lung, and esophageal cancer comparing to control subjects [29]. In addition to muscular OS, systemic OS seems to be exacerbated specifically after the onset of cachexia, since ROS production in the blood was greater in cachectic patients with lung cancer, comparing to noncachectic patients with lung cancer [30]. Mantovani and coworkers established a direct association between systemic OS and the performance status of cachectic patients. They found that the high blood levels of ROS were somehow associated with increased fatigue, decreased autonomy, and elevated concentrations of proinflammatory cytokines [31]. Liver biopsies from cachectic patients with esophageal, lung, and kidney carcinomas also revealed an increase in hepatic MDA-protein adducts [32]. Interestingly, the inflammatory profile associated with cachexia reduced the hepatic drug clearance in cancer patients* via* depressing the expression of cytochrome P450 (CYP) in liver, namely, CYP3A [33, 34]. This could prolong the blood exposures of drugs and increase toxicity risk in patients undergoing chemotherapy. Furthermore, CYP3A is involved in the metabolism of several opioid analgesics used to alleviate cachexia symptoms; thus the decrease in CYP3A expression and activity could also affect the management of pain in cancer cachectic patients [33, 34].

**Figure 1:**
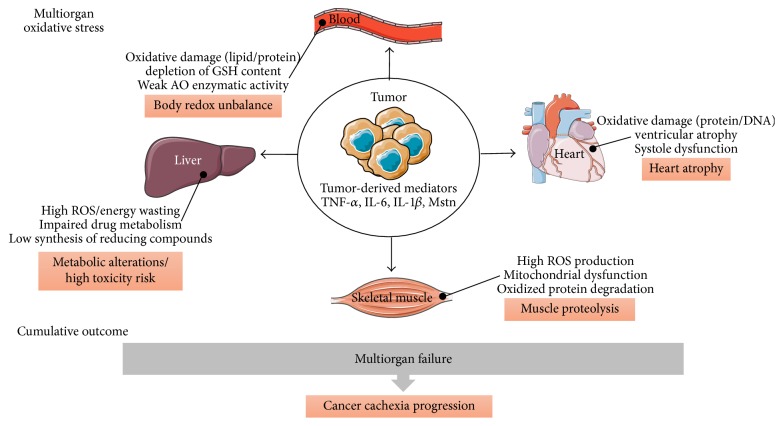
The central role of tumor in the development of oxidative stress at multiple organs during cachexia. Tumor is the main responsible factor for the development of OS at different organs and the consecutive disruption of their vital functions. Indeed, chemicals released by tumor in the systemic circulation can reach multiple destinations like heart, muscle, and liver. For example, TNF-*α* and IL-6 can induce anorexia, leading to inadequate synthesis of reducing compounds like NADPH in the liver. Additionally, IL-6, TNF-*α*, and myostatin (Mstn) upregulate the activity of ROS-producing enzymes within heart/skeletal muscles, leading to the activation of several catabolic pathways and muscle proteolysis. As a direct result, heart/skeletal muscles are atrophied, oxidative injuries accumulated, and antioxidant (AO) defense becomes inefficient, giving way to multiorgan failure and cancer cachexia evolution.

*Animal Studies*. OS was also reported in skeletal muscle and other tissues of cachectic animals. For example, protein carbonylation and lipid peroxidation adducts, namely, 4-Hydroxynonenal (4-HNE) and MDA, were increased in the* gastrocnemius* (Gas) muscle of rats bearing Yoshida AH-130 hepatoma tumor [35]. In our own laboratory, we have shown that implantation of colon 26 (C26) cells into BALB/c mice induced cachexia and skeletal muscle atrophy. Cachectic C26 mice exhibited a net augmentation in protein carbonyls and 4-HNE content within plasma, without any change in skeletal muscle. The absence of muscular oxidative damage in our model could be attributed to the upregulation of catalase expression, exclusively, in atrophied muscles [17]. Other experimental studies have also shown that mice bearing Walker 256 and MAC13/16 tumors developed cardiac cachexia in response to DNA and/or protein oxidative damage in heart tissues [20, 36]. Additionally, mice bearing C26 tumor exhibited an upregulation in gene-specific inflammation within heart and manifested a reduction in cardiomyocytes diameter, loss of ventricular mass, and systolic dysfunction [37–39]. Indeed, the treatment of primary rat cardiomyocytes with the conditioned milieu of C26 cells induced atrophy, increased mitochondrial stress, and triggered an aberrant lipid oxidation metabolism [39]. These data suggest that tumor-borne factors promote cardiac dysfunction in cachexia. Besides heart atrophy, cachexia was able to suppress the expression of CYP in liver of mice [40] and increase ROS production ~12-fold in liver of cancer bearing rats [21]. Therefore, tumor-derived factors are mainly responsible for the deregulation of body redox homeostasis and the development of OS that might lead to multiorgan failure and enhance cachexia progression (Figure 1). As skeletal muscle wasting is a key feature of cancer cachexia, hereafter, we will focus and describe main ROS-dependent mechanisms involved in muscle proteolysis and the interplay between tumor and muscle.

## 3. ROS Production and Inflammation: Causality Link and Principal Mechanisms

### 3.1. Tumor-Derived Chemicals

*Clinical Studies*. Proinflammatory cytokines, transforming growth factor-beta (TGF-*β*) family ligands, and other tumor-specific mediators like proteolysis-inducing factor (PIF) are expressed and released continuously by tumor cells [41]. Once in bloodstream, these mediators can easily reach skeletal/cardiac muscles and promote ROS formation by binding to their cognate receptors expressed on the surface of muscle cells [42]. Basically, ROS promote muscle wasting and cachexia progression through the activation of three main catabolic pathways: ubiquitin proteasome system (UPS), autophagy lysosome pathway, and calcium-dependent calpain pathway. Elevated levels of tumor necrosis factor-*α* (TNF-*α*), interleukin-6 (IL-6), and PIF were reported in biological fluids (e.g., blood and urine) of patients experiencing cachexia [28, 29, 31, 43]. In pancreatic cancer patients, systemic inflammation was correlated with the activation of proteasome system in skeletal muscle [44]. Interestingly, gastric cancer patients with no weight loss exhibited an increase in calpain activity in the* rectus abdominis* muscle, without any change in the expression of key components of the UPS,* MuRF-1,* and* MAFbx* [45]. On the other hand, proteasome activity was significantly higher within* rectus abdominis* of weight-losing patients with advanced stage of gastric cancer [46]. These findings may emphasize the fact that calpains are activated earlier during cachexia related to gastric cancer, before substantial weight loss and hypercatabolism of skeletal muscle by the UPS. In other cancers, such as esophageal cancer, muscle proteolysis seems to be dependent on the activities of lysosomal proteases, cathepsins B and L, indicating a possible involvement of autophagy in the pathogenesis of muscle wasting clinically [47]. Together, these data may suggest that the activation of a specific catabolic pathway depends on the type of cancer and, therefore, the nature of circulating humoral factors. For example, excessive skeletal muscle loss and cachexia related-death culminate in patients with colorectal, pancreatic, and lung cancer, whereas those with breast, sarcomas, and non-Hodgkin's lymphoma are usually spared [48].

**Figure 2:**
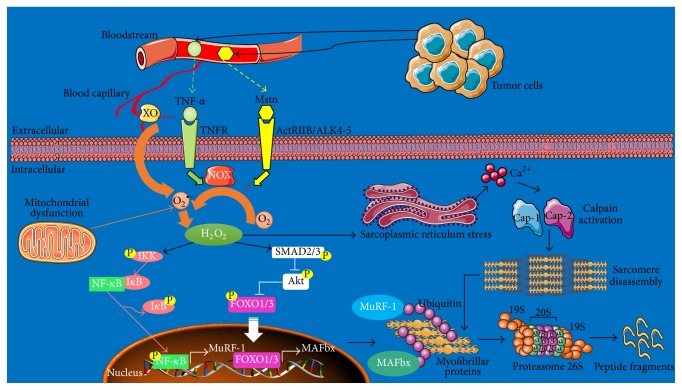
Role of ROS as a second messenger in the activation of proteolysis pathways. Tumor cells produce great amounts of proinflammatory cytokines and TGF-*β* family ligands, such as TNF-*α* and Mstn, respectively. Once in bloodstream, these mediators can easily reach skeletal muscle and activate several catabolic pathways, by signaling through their specific receptors. TNF-*α* induces the activation of NOX found in muscle fibers. The elevated activity of NOX and XO (XO is usually located within blood capillaries irrigating muscle) during cachexia is responsible for the great production of anion superoxide (O_2_^∙−^) molecules, which are rapidly converted into hydrogen peroxide (H_2_O_2_). Accumulation of H_2_O_2 _within muscle fibers induces sarcoplasmic reticulum stress and the subsequent massive release of calcium (Ca^2+^) ions. The increase of intracellular Ca^2+^ concentrations activates calpains 1 and 2 (Cap-1 and Cap-2), which in turn promote sarcomere disintegration and myofibrillar proteins liberation. H_2_O_2 _can activate IkB kinase (IKK) or SMAD3, leading to the phosphorylation of IkB and the dissociation of the NF-kB/IkB complex. Subsequently, NF-*κ*B is released and ready to translocate into the nucleus. Additionally, P-SMAD2/3 transducers remove the sustained inhibitory phosphorylation of P-FOXO1/3 exerted by Akt and, therefore, allow its nuclear accumulation. Upon their entry into the nucleus, P-NF-*κ*B and FOXO1/3 promote the transcriptional activation of MURF-1 and MAFbx, respectively. Then, MURF-1 and MAFbx tagged myofibrillar proteins with polyubiquitin chains to undergo proteolytic processing by the proteasome core (adapted from [27]).

*Animal and Cell Culture Studies*. The presence of high ROS levels within muscle cells alters the function of numerous organelles, which in turn may induce muscle dysfunction and foster the degradation process of sarcomeric proteins [49]. For example, hydrogen peroxide (H_2_O_2_) induces endoplasmic reticulum stress leading to myoplasmic calcium (Ca^2+^) accumulation and, therefore, the activation of calpains [50, 51]. Calpains promote the disintegration of sarcomere structure and liberation of actin/myosin filaments for the proteasome machinery [49]. In parallel, ROS mobilize various transcriptional factors directly involved in the regulation of genes related to catabolic pathways. A previous study demonstrated that nuclear factor-*κ*B (NF-*κ*B) was rapidly activated by H_2_O_2_, following treatment of C2C12 muscle cells with TNF-*α* [10]. We have also shown that circulating levels of TNF-*α* were increased in cachectic mice bearing colon tumor and coincided with a greater phosphorylation of the NF-*κ*B (p65) subunit, within atrophied muscles [17]. The nuclear accumulation of NF-*κ*B promotes the transcriptional upregulation of muscle-specific E3 ubiquitin-ligases,* MuRF-1* and* MAFbx*, which in turn tag myofibrillar proteins (i.e., myosin) with polyubiquitin chains for proteasome processing [52]. NF-*κ*B also induces the expression of proteasome subunits and proinflammatory cytokines, like IL-6, thereby maintaining a vicious circle [53, 54]. In the same way, IL-6 was described as a potent activator of signal transducer and activator of transcription 3 (STAT3), which controls the activation of UPS-dependent elements [55] and both expression and activity of cathepsins B and L in the atrophied muscles [11]. Accordingly, the blockade of IL-6 with a specific antibody attenuated cachexia severity and muscle wasting in C26 mice [11]. High levels of Forkhead box (FOXO) were also reported in muscles of cachectic animals [56]. Thus, emerging pieces of evidence suggest a possible role for ROS in controlling the transcriptional activity of FOXO [57], which is a master regulator of a plethora of genes related to the UPS and autophagy mechanisms such as autophagosome biosynthesis and autophagosome-lysosome fusion [58].  Figure 2 illustrates the role of ROS as a second messenger in the activation of main catabolic pathways within skeletal muscle, in response to* bona fide* tumor cytokines.

### 3.2. Eicosanoids

*Clinical Studies*. Lipoxygenase (LOX) and cyclooxygenase (COX) are two enzymes producing potent inflammatory mediators called eicosanoids. Brain, skeletal muscle, and some tumor types express both enzymes and specific receptors for eicosanoids [59]. Three isoforms are identified for LOX (5-LOX, 12-LOX, and 15-LOX) and two for COX (COX-1 and COX-2). Arachidonic acid (AA) constitutes the main substrate for LOXs, to produce leukotriene (LT) and hydroxyeicosatetraenoic (HETE) acid, and for COX in the synthesis process of prostaglandin (PG) and thromboxane (TAX) [59]. Clinically, six-week selective inhibition of COX-2, using celecoxib, reduced the severity of cachexia symptoms in lung cancer patients through improving muscle strength and lowering the circulating levels of C-reactive protein (CRP) (marker of systemic inflammation) [60]. Treatment with celecoxib, during four months, was also effective in attenuating the blood levels of TNF-*α*, decreasing fatigue, and increasing lean body mass in patients with ovary, pancreas, and colorectal cancer [61]. Similar findings were obtained from patients with head and neck cancer treated with celecoxib for three weeks [62]. These clinical results suggest a potential role for COX-2 in promoting chronic inflammation observed in cancer cachexia and the related muscle wasting. Nonetheless, there is a lack of information concerning the regulation of LOX in clinical cancer cachexia.

*Animal and Cell Culture Studies*. In experimental cancer cachexia, the inhibition of 5-LOX using CV-6504 attenuated tumor growth and cachexia progression in animals bearing MAC16 and MAC26 adenocarcinoma [63, 64]. Additionally, the inhibition of COX-1/2 using indomethacin or COX-2 with NS 398 rescued muscle wasting related to Lewis lung carcinoma (LLC) or C26 tumor but had no effect on muscle loss in mice bearing B16 melanoma [65, 66]. Importantly, the preservation of muscle mass was due to the regression of tumor growth and reduction in circulating eicosanoids and IL-6 amounts as well as the decrease in TNF-*α* receptor-1 levels within Gas muscles [65–67]. These findings indicate that the crosstalk between tumor and skeletal muscle and the resulting catabolic response depend largely on LOX/COX metabolites and cytokines. Importantly, these eicosanoids could mediate the catabolic actions of tumor-derived cytokines through activating a number of ROS-producing enzymes and increasing ROS generation [68, 69]. For example, in response to specific tumor factors, high levels of 15-HETE could be produced to enhance ROS production and protein degradation within muscle cells [69]. Therefore, we suppose that “cytokines-eicosanoids-ROS-muscle catabolism” is the main axis through which tumor induces muscle loss during cachexia.

## 4. Main Sources of ROS in Cancer Cachexia

### 4.1. Elevated Activity of ROS-Producing Enzymes

#### 4.1.1. Xanthine Oxidase

*Clinical Studies*. In normal conditions, the highest levels of xanthine oxidoreductase (XOR) activity are present in intestine of mammals, contrary to muscles tissues in which XOR activity is very low [70]. XOR exists in two interconvertible forms that are xanthine dehydrogenase (XDH) and xanthine oxidase (XO). In several pathological states, the presence of proinflammatory cytokines promotes the cleavage of XDH to XO, which instead uses molecular oxygen to catalyze the hydroxylation of hypoxanthine to xanthine and, then, to uric acid, producing ROS, mainly anion superoxide (O_2_^∙−^) and H_2_O_2_ [71]. The role of XO was mainly addressed in cancer patients, regardless of the stage of cachexia. Herein, we will describe a number of these studies, in an attempt to elaborate a hypothesis about the eventual role of XO in cancer cachexia. Studies in humans demonstrated an increase in blood XO activity in patients with non-small-cell lung carcinoma (NSCLC), small-cell lung carcinoma (SCLC), head and neck carcinoma, and liver cancer compared to control patients [72–74]. The activity of XO was in most cases positively correlated with prooxidant parameters in blood samples (i.e., lipid peroxidation adducts) [72, 74]. An elevated activity of XO was also noted in the plasma of patients with acute lymphoblastic lymphoma, while patients with cervix cancer exhibited a low activity of XO [75]. However, there is a lack of information concerning the modulation of XO activity in the skeletal muscle of cancer patients. Based on clinical data, it appears that the activity of XO in blood is most likely elevated in cancer patients and, therefore, its inhibition could be beneficial. Accordingly, accumulating evidences from animal studies globally support an involvement of XO in the pathophysiology of cancer cachexia. Thus, the activity of XO is expected to increase in cachectic cancer patients, but clinical studies are still needed to confirm such hypothesis.

*Animal Studies*. In the experimental models of cancer cachexia, rats bearing Yoshida tumor and mice bearing MAC16 adenocarcinoma, the activity of XO was elevated in skeletal and/or cardiac muscles and correlated with an increase in muscle oxidative damage [20, 76–78]. Although XO is not usually present at high levels within skeletal muscle, the hyperactivation of XO during cachexia could be explained by an increase in the cleavage of XDH to XO [76]. The small number of studies that addressed the role of XO in cachexia-induced muscle wasting demonstrated that targeting XO with selective inhibitors such as allopurinol (4 and 40 mg/kg/day), oxypurinol (4 and 40 mg/kg/day), and febuxostat (5 mg/kg/day) can reduce body weight loss and skeletal muscle/heart atrophy [76–78]. The molecular mechanisms behind these beneficial effects of XO inhibition are mainly (1) attenuation of oxidative damage within skeletal muscle, (2) inhibition of DNA binding potential of transcription factors like NF-*κ*B and STAT-3, (3) reduction of proinflammatory cytokines expression, (4) decrease in the expression of key components of the UPS (e.g., ubiquitin, MuRF-1), and (5) reinforcement of protein synthesis pathways (e.g., Akt activation) [76–78]. Preliminary results from our laboratory indicate that treatment of C26 tumor-bearing mice with allopurinol (50 mg/kg/day) partially prevented the decrease in* extensor digitorum longus* (EDL) muscle fiber diameter but failed to improve total body and skeletal muscle weight loss (Table 1). This could be attributed to the fact that protein carbonyls and 4-HNE content, although present in plasma, were absent in skeletal muscle, while in the study of Springer et al., showing improvement of muscle mass after allopurinol administration, the content of protein carbonyls was greater within wasted muscles and significantly decreased in response to allopurinol [76]. Additionally, allopurinol failed to attenuate systemic oxidative damage in C26 mice. This may indicate that XO is not a primary actor in the pathogenesis of muscle wasting related to C26 tumor.

#### 4.1.2. Nicotinamide Adenine Dinucleotide Phosphate Oxidase

*Clinical Studies*. The family of nicotinamide adenine dinucleotide phosphate oxidase (NOX) produces both O_2_^∙−^ and H_2_O_2 _[79]. Seven isoforms have been identified to produce ROS, among which NOX-4 produces H_2_O_2_ and NOX-1, NOX-2, and NOX-5 generate O_2_^∙−^ [79]. In conditions evoking chronic inflammation, which is the case of cachexia, high amounts of ROS originating from NOX could negatively influence gastrointestinal and pancreatic cancer development [80]. Clinically, the expression of NOX-1 and NOX-4 in tumor was associated with poor survival and cancer relapse [81, 82]. Another isoform, NOX-5, was also found to be overexpressed in numerous cancers, including colon, melanoma, breast, lung, and prostate cancer [80]. However, the role of NOX in cancer and cancer cachexia has not been addressed in depth clinically and further studies are needed to establish its exact role. At the moment, it seems that the expression of NOX within tumor is associated with cancer progression [80]. 

*Animal and Cell Culture Studies*. TNF-*α*, IFN-*γ*, PIF, and Angiotensin-II (Ang-II) are known to induce ROS production* via* the activation of NOX [83, 84]. In a model of Ang-II-infused mice, the high formation of O_2_^∙−^ levels within muscles upregulated the expression of E3-ligases MuRF-1/MAFbx and promoted proteasome-mediated proteolysis [83]. This elevated production of O_2_^∙−^ was NOX-dependent, since its blockade with a specific inhibitor, apocynin, partially prevented atrophy. Contrariwise, it is thought that the enhanced O_2_^∙−^ formation within skeletal muscle of cachectic mice bearing MAC16 tumor was due to an aberrant antioxidant response rather than an increase in NOX activity [85]. PIF was able to promote phospholipase A2-catalyzed release of AA from membrane phospholipids. The conversion of AA into 15-HETE, by 15-LOX, promoted NOX-induced O_2_^∙−^ production and the subsequent activation of NF-*κ*B/UPS proteolysis pathway in muscle cells [69]. In addition to skeletal muscle, LOX/NOX signaling is one of the prosurvival mechanisms that makes pancreatic cancer cells unresponsive to anticancer treatments [86]. Since NOX controls the activation of various downstream kinases that play an essential role in proliferation, differentiation, and inflammation, the silencing of NOX isoforms, especially NOX-4, could provide a particular therapeutic interest to limit cancer cells proliferation and reduce the magnitude of muscle degradation.

#### 4.1.3. Nitric Oxide Synthase

*Clinical Studies*. Nitric oxide (NO) is a free radical produced enzymatically by NO synthase (NOS) from l-arginine. NOS exists in three different isoforms: Type I NOS and Type III NOS (eNOS), expressed constitutively in the skeletal muscle, and Type II NOS also called inducible NOS (iNOS) expressed exclusively in the presence of proinflammatory cytokines such as TNF-*α*, IFN-*γ*, and IL-1 [87]. At high concentration, NO can induce nitrosative stress through reacting with O_2_^∙−^ and, subsequently, producing elevated levels of peroxynitrite molecules extremely injurious for muscle [5]. Nitrotyrosine is usually used as a biomarker to evaluate the level of nitrosative damage. Today, it is admitted that the arginine/NO metabolism is altered in cachectic patients and responsible for the inhibition of protein synthesis and activation of proteolysis [88]. High NO levels were found in plasma of patients with gastric cancer comparing to those without cancer [89]. Cachectic patients with advanced stages of cancer presented a greater NO production, nitrotyrosine content, and iNOS expression in skeletal muscle tissues, comparing to noncachectic subjects [29, 87]. Importantly, iNOS was also found to be expressed in tumor tissues of patients and its expression correlated positively with tumor size and aggressiveness, especially in breast and colorectal cancer [90]. 

*Animal and Cell Culture Studies*. In cachectic nude mice overexpressing TNF-*α* gene, the NOS system was activated and responsible for the disruption of D-Jun/myogenin-complex binding to the myosin creatinine phosphokinase enhancer (MCK-E) box, leading to muscle atrophy and dedifferentiation [91]. The inhibition of NOS, by nitro-l-arginine, prevented weight loss and muscle wasting in TNF-*α*-treated animals [91]. Apoptosis is one of the mechanisms that could be involved in muscle atrophy. Caspase-3, jointly with calpains, mediates the dissociation of actinomyosin complex, making myofilaments susceptible to UPS degradation [49]. Interestingly, a link between iNOS and apoptosis activation has been suggested, since the administration of IL-15 to cachectic rats inhibited apoptosis by disturbing TNF-*α* signaling and the resulting NO formation [92]. In C2C12 cells, TNF-*α* and IFN-*γ* were able to induce the activation of NF-*κ*B and its downstream target iNOS [93]. The activation of TNF-*α*/NF-*κ*B/iNOS pathway was efficient to promote the degeneration of muscle* via* stimulating the loss of proteins playing a key role in muscle cell proliferation and differentiation such as MyoD [93]. These compelling evidences indicate that selective inhibition of iNOS could decelerate cachexia progression in cancer.

### 4.2. Mitochondrial Dysfunction

**Figure 3:**
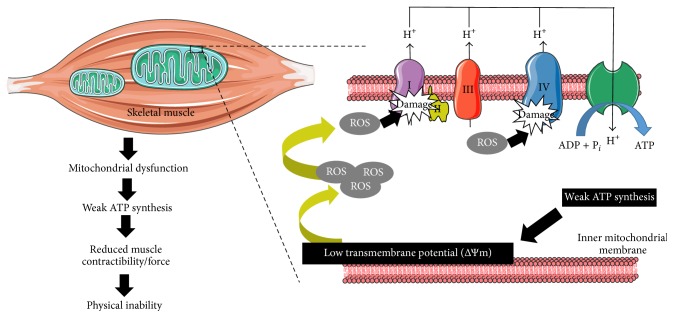
Mitochondrial dysfunction in wasted muscles. High ROS amounts present within atrophied muscles impair mitochondrial ATP synthesis by causing direct oxidative damage in the electron transport chain. This weak ATP production leads to a low mitochondrial transmembrane potential, allowing mitochondria to produce very excessive rates of ROS, thereby maintaining the vicious circle. All these events contribute to muscle wasting development through impairing muscle contractibility and ability to generate force.

A scarce number of preclinical studies have addressed the mitochondrial events that occur within skeletal muscle during cancer cachexia but data from humans are still lacking. Mitochondrial dysfunction and altered mitochondrial plasticity are a primary source of ROS generation in cachexia. ROS exert direct deleterious effects on mitochondrial respiratory chain (MRC) complexes (i.e., complexes I, II, and IV) by decreasing their activities in skeletal and respiratory muscles of cachectic mice [94]. Thus, it makes sense that ROS-mediated MRC dysfunction could lead to impaired oxidative phosphorylation and low adenosine triphosphate (ATP) synthesis. In numerous animal models of cachexia related muscle wasting, skeletal mass degradation was associated with a decrease in respiratory chain activity and low ability of wasted muscles to synthetize the required ATP [95, 96]. Indeed, treatment of C2C12 muscle cells with LLC conditioned culture medium (rich in proinflammatory mediators) increased ROS production and reduced ATP production [97]. These disruptions in respiratory chain function were mainly due to mitochondrial loss (i.e., mitophagy), structural abnormalities (i.e., giant mitochondria), and increased uncoupling proteins (UCPs) expression, namely, UCP2 and UCP3 [98, 99]. As depicted in Figure 3, a weak ATP production leads to a low mitochondrial transmembrane potential [100], allowing mitochondria to generate excessive amounts of ROS potentially damaging for mitochondria membrane and muscle. Thus, there is a ROS-ATP-ROS loop during cachexia. ROS primarily produced in response to inflammatory stimuli disturb the MRC function within muscle, leading to a decreased ATP formation [97]. This poor ATP level is a favorable condition for high mitochondrial ROS production [100], thereby maintaining the vicious circle. The mitochondrial energetic inefficiency and the subsequent accumulation of oxidative insults may impede the capacity of muscle to generate sufficient force and ensure basic physical needs [101]. This ROS-dependent mechanism observed in skeletal, cardiac, and respiratory muscles may in part explain the increased fatigue and decreased autonomy observed in cachectic individuals with advanced stages of cancer.

### 4.3. Defective Antioxidant Responses

*Clinical Studies*. In addition to the above-mentioned sources of ROS, the loss of antioxidant counterbalance and control can exacerbate OS in cancer cachexia. At the systemic level, SOD activity was upregulated in patients, with stage II to stage IV cancer, presenting a good performance status, while SOD activity decreased along with GPx activity in cachectic patients with compromised physical performance at stage IV [31], indicating that high grade cancer and poor muscle strength are, most likely, associated with a weak enzymatic antioxidant activity. Furthermore, patients bearing breast or colon cancer displayed a low blood level of reduced glutathione (GSH) [102]. The decrease in GSH content may be due to a decrease in the available substrates needed for GSH synthesis. In fact, glucose plays a pivotal role in the synthesis of compounds with high reducing potential, like NADPH, through the pentose phosphate pathway. NADPH is required for (1) the reduction of GSH disulphide (GSSG) to GSH, by the GSH reductase, and (2) formation of active catalase tetramers [103]. The perturbations in glucose metabolism and reduced nutrients supply, due to symptoms such as anorexia and vomiting, can lead to an inadequate synthesis of reducing compounds and, therefore, may explain the GSH deficiency observed in cachectic individuals [104]. 

*Animal and Cell Culture Studies*. Treatment of C2C12 cells with TNF-*α* caused a net decrease in GSH content, which coincided with elevated ROS generation and atrophy development [105]. In line with these* in vitro* findings, both expression and activity of SOD and GPx decreased in the skeletal/cardiac muscles of cachectic mice [20, 85, 106]. On the other hand, other experimental studies found that the expression of SOD was upregulated within atrophied skeletal muscles [28, 76]. We have reported an increase in catalase expression within skeletal muscle of cachectic mice without any change in CuZnSOD and MnSOD expression [17]. Nonetheless, studies that denoted an increase in SOD activity have also demonstrated an increase in OS profiles, suggesting that SOD activation was inefficient and insufficient to antagonize muscular and systemic OS. An accumulation of high H_2_O_2_ rates due to the elevated SOD activity might explain this paradox. However, data available from the literature strongly suggest that the decrease of muscle and blood GSH content, GSH/GSSG ratio, and GPx activity occur during cancer cachexia related muscle wasting. In addition to the involvement of ROS in the pathophysiology of muscle wasting, these species directly regulate the growth/death balance within tumor itself and several ROS-dependent mechanisms have been unveiled (see Section 6).

## 5. The Dual Role of ROS in Tumor

*Clinical Studies*. The role of ROS in cancer has been previously discussed in detail [107, 108]. Contrary to skeletal muscle in which lessening oxidative damage is relatively advantageous during cachexia, the reduction of tumor OS could be deleterious in some cases. ROS play a dual role within tumor; on the one hand they have the ability to promote tumorigenesis and vascularization [109]. On the other hand they can induce DNA damage, cell cycle arrest, and apoptosis [8]. This double-faced role of ROS was underscored clinically. Accordingly, high levels of ROS were detected in human hepatocellular cancerous tissues comparing to normal adjacent tissues [110]. It is though that ROS accumulation could promote cancer progression* via* the activation of several transcriptional factors, including FOXO6, regulating the expression of cell cycle genes (i.e., p27 and cyclin-D1) [110]. Thus, in this case, the inhibition of ROS could be beneficial to slow cancer growth. But ROS can also generate intracellular signals that stimulate cell death, and new anticancer targeted therapies using encapsulated nanoparticles (i.e., HSP90 inhibitor) mainly rely on the generation of excessive ROS amounts to promote apoptosis and improve cancer care [111]. Therefore,* can some tumor types also benefit from antioxidants?* Indeed, the impact of antioxidant supplementation on both tumor progression and regression was mainly addressed in animals. 

*Animal Studies*. Accumulating evidence from high-quality studies indicates that antioxidants could be detrimental in cancer bearing mice. Piskounova et al. elegantly demonstrated that high ROS levels protected against melanoma metastasis in NSG mice, since metastatic cells presented a lower ROS generation comparing to subcutaneous nonmetastatic tumor [112]. Similar findings were obtained by Le Gal et al. showing that administration of antioxidants enhanced the invasive potential of melanoma tumors without affecting proliferation [113]. Thus, in addition to the modulation of cell cycle, ROS control tumor behavior through the regulation of cytoskeletal proteins involved in cell migration and invasion [113]. We have recently shown that the reduction of tumor OS in cachectic mice bearing C26 colon cancer accelerated proliferation [17]. Contrariwise, in rats bearing AT-1 prostate cancer, the inhibition of OS decreased tumor oxidative damage and proliferation [114], indicating that the reduction of OS could either enhance or slow tumor proliferation and progression depending on tumor type and localization. In other words, the redox state of tumor is an important factor that could swing the balance of a given antioxidant treatment towards the beneficial or harmful side. Thereafter, in some cases the inappropriate use of antioxidants could promote tumor growth through decreasing ROS production and oxidative damage. A direct consequence of the enhanced tumor growth is an increase in the circulating levels of tumor-derived mediators and the subsequent cachexia development.

## 6. Antioxidant Supplementation in Cancer Cachexia: Impact and Molecular Mechanisms

### 6.1. Antioxidant Vitamins and Carotenoids

*Clinical Studies*. No previous studies have addressed the role of individual vitamins in cancer cachexia. Antioxidants were usually given as a mixture containing vitamins, polyphenols, and other antioxidant compounds [13, 14]. Most intervention studies with antioxidant vitamins performed on cancer patients did not explore the concept of cachexia or take into account cachexia staging criteria to select patients. However, as mentioned above, tumor occupies a central role in the development of cachexia; thus, in a first step it could be helpful to draw a global view about the impact of vitamins on cancer itself with the aim of better using these products in cancer cachexia. Data available from clinical studies suggest a lack of convincing evidence concerning the beneficial effects of vitamin supplementation in cancer patients [115]. The systematic review and meta-analysis of Bjelakovic et al. incorporated the results of 14 randomized trials and concluded that high-doses of vitamin A/E and *β*-carotene were associated with increased mortality in patients with gastrointestinal cancer [18, 116]. Accordingly, the meta-analysis from Pais and Dumitraşcu indicated that the combination of *β*-carotene with vitamin E could increase mortality in patients with colorectal cancer [117]. Interestingly, this increase in mortality seems to be more pronounced when doses of vitamin E exceeded 134 mg/day [118]. Thus, if antioxidant vitamins are problematical in some cases,* how should we improve the use of such compounds?* In other words,* what is the factor that will provide eligibility for a cancer patient to undergo antioxidant supplementation?* The response seems to be provided by the randomized double-blinded trial “SUVIMAX.” At the baseline, healthy men enrolled in the study exhibited a low blood antioxidant status compared to healthy women, because of the reduced intake of fruits/vegetables often observed in men's alimentary habits [119]. After eight years of daily supplementation with complements including vitamin C/E and *β*-carotene at nutritional doses, men presented a reduced risk of 31% to prostate cancer, while women with adequate antioxidant status at the baseline developed an increased risk of 67% to skin cancer [119], indicating that only individuals with particular antioxidant deficiencies will benefit from supplementation in terms of cancer prevention. This conclusion, although obtained in disease-free subjects, could be logically transposable to cancer cachectic patients.

The small number of intervention studies with antioxidants conducted on cachectic patients with head and neck, ovary, colorectal, lung, and breast cancer supports the evidence that vitamins in combination with other antioxidants could be beneficial in patients with weak blood antioxidant activity and high ROS levels [120]. Intriguingly, a previous study has shown that patients with lung cancer exhibited low blood levels of vitamin E comparing to controls, but the depletion of vitamin E was more pronounced in cachectic patients [30]. This may indicate that even in the same type of cancer the doses of vitamins must be adapted taking into account the presence or absence of cachexia. Recently, the French speaking society of clinical nutrition and metabolism (SFNEP) has discouraged the use of *α*-tocopherol and *β*-carotene for patients with esophageal and head and neck cancer without diagnosed deficiency [121]. The SFNEP has also stressed out the negative impact of a high-dose and long-term antioxidant vitamins administration on the effectiveness of radio/chemotherapy [121]. Accordingly, supplementation with vitamin E and *β*-carotene increased cancer recurrence and overall mortality in head and neck cancer patients undergoing radiotherapy [122]. According to the review of Harvie it seems that the association of antioxidant vitamins with radiotherapy reduces its anticancer potential [24]. However, there is a lack of evidence concerning the combination of vitamins with chemotherapy. At the moment, the best way to provide an effective nutritional support for cachectic cancer patients is to determine and adapt vitamins doses on a patient-by-patient basis. The supplementation must target cachectic patients exhibiting reduced blood levels of vitamins A, C, and E, *β*-carotene, and lycopene [123, 124]. 

*Animal Studies*. Experimental models of cancer permitted us to understand some of the mechanisms borrowed by vitamins to induce their deleterious effects. The principal and commonly described mechanism was* via* lessening oxidative damage and ROS-induced apoptosis in tumor. In preclinical studies vitamin E, in the form of *α*-tocopherol, was the most used antioxidant vitamin given its kinetic ability to scavenge certain free radicals (*k* ~ 10^5^–10^6^ M^−1^ s^−1^) [125]. Vitamin E (100–500 mg/kg) accelerated lung cancer progression in mice through decreasing ROS production and oxidative damage to DNA (i.e., 8-oxoguanine) within tumor [126]. Moreover, vitamin E enhanced the proliferation of lung cancer cells by reducing the expression of the redox-dependent protein p53, which is responsible for cell cycle arrest and apoptosis induction [126]. Vitamins C (8 mg/kg) and E (40 mg/kg) were also able to attenuate the anticancer activity of cisplatin combined with an omega-3 enriched diet, by decreasing lipid peroxidation in lung tumor tissue [127]. Since muscle wasting is a key feature of cancer cachexia, most experimental studies have attempted to use antioxidants with the aim of preventing OS in muscle but did not take into account the redox status of tumor. Although vitamin E was able to attenuate skeletal muscle proteolysis in unloaded mice by reducing muscular OS [128], the use of a mixture containing nutritional doses of vitamins A (0.06 mg/kg), C (11.53 mg/kg), and E (1.73 mg/kg) selectively reduced oxidative damage in C26 tumor and promoted its growth but exacerbated OS within skeletal muscle [17]. Remarkably, these findings may indicate that the use of antioxidant vitamins is more complicated in cachexia-related muscle wasting due to the ambivalence of OS between skeletal muscle and tumor.

### 6.2. Polyphenols

*Clinical Studies*. Cachectic patients with head and neck, colon, and lung cancer presented higher ROS levels and low enzymatic antioxidant activity in the blood compared to healthy individuals [129]. Their supplementation with an antioxidant formula containing polyphenols (300 mg/kg) partially reduced systemic OS and improved performance status [15]. Green tea polyphenols (474 mg/day) also attenuated ROS levels in plasma of patients with liver cancer undergoing arterial infusion chemotherapy [130]. A short-term treatment of prostate cancer patients with green tea extracts reduced the circulating levels of prostate-specific antigen (PSA) and vascular endothelial growth factor (VEGF), supporting a potential positive role for polyphenols in cancer prevention and treatment [131]. 

*Animal Studies*. Epigallocatechin-3-gallate (EGC-3-G) and theaflavin-3,3′-digallate, found in green and black tea, respectively, were effective in reducing skeletal muscle atrophy caused by cachexia, through inhibiting TNF-*α*-mediated activation of NF-*κ*B system [132, 133]. Rats bearing Walker 256 tumor receiving daily intraperitoneal (IP) quercetin injections (10 mg/kg) presented tumor regression and prolonged survival [134]. These beneficial effects of quercetin on tumor growth could be attributed to its antiangiogenic properties, as evidenced by the inhibition of VEGF production in liver extracts [134]. Furthermore, oral quercetin supplementation (25 mg/kg) improved the musculoskeletal function and altered IL-6 production in cachectic Apc^Min/+^ mice, independently of tumor burden [135]. Accordingly, the phosphorylated levels of STAT3 (downstream effector of IL-6) were decreased in skeletal muscle of Apc^Min/+^ mice supplemented with quercetin, while the phosphorylation status of NF-*κ*B remained unchanged [135]. Resveratrol, abundantly found in the skin of grapes, peanuts, and pines, seems to exert its antiwasting effects* in vivo* largely depending on tumor type and the route of administration. Oral resveratrol (200 mg/kg) therapy reduced muscle loss through impairing the DNA binding activity of NF-*κ*B (p65) subunit in both skeletal and cardiac muscles of mice bearing C26 tumor, without influencing tumor growth [136], whereas IP resveratrol injection failed to ameliorate muscle wasting in mice bearing LLC (1 mg/kg) or Yoshida AH-130 (5 and 25 mg/kg) tumor [137]. Although most of these studies found that polyphenols positively affected muscle mass and function, there is a lack of evidence concerning their effects on tumor growth. Globally, tumor weight was the sole parameter used to underscore tumor regression; this data must be consolidated by performing direct analysis on tumor proliferation (e.g., Ki-67, mitotic index), apoptosis, OS, and local inflammation.

### 6.3. Multimodal Therapy

Since the etiology of cachexia is multifactorial, antioxidants alone cannot fully prevent or reverse muscle atrophy during cachexia. Thus, treatments should be multidimensional to alleviate cachexia symptoms and overcome related sufferance. However, with respect to the topic and aims of the present review, we will discuss in this paragraph only studies that have integrated antioxidants in their treatment arms against cachexia (Table 2). In a randomized phase III study, treatment of gynecological cancer patients with antioxidants, namely, *α*-lipoic acid and carbocysteine, combined with megestrol acetate (MA, appetite stimulant) and L-carnitine (antioxidant properties) decreased fatigue, circulating TNF-*α* concentrations, and ROS blood levels, whereas MA alone failed to induce any significant changes in all these parameters [12]. Decidedly, the pioneer work of Mantovani's team clearly indicates that the supplementation of cancer cachectic patients with a cocktail of antioxidants, including polyphenols, vitamins, and cysteine-containing compounds, alone or associated with drugs like MA, L-carnitine, and thalidomide (immune-modulatory function), increased the activity of GPx and reduced ROS levels in blood [15, 138]. Additionally, this combination regimen can effectively ameliorate lean body mass and the performance status in cachectic patients, as assessed by the European cooperative oncology group (ECOG) scale. These clinical positive outcomes could be attributed to the presence of high ROS amounts and the low activity of antioxidant enzymes in blood samples at the baseline. Consistent with this interpretation, the study of Block et al. showed that supplementation with high-doses vitamins C (1000 mg) and E (800 UI) during two months reduced the plasmatic levels of isoprostane (marker of lipid peroxidation), only if it was superior to 50 *μ*g/mL [139]. This indicates the existence of plasmatic critical threshold values for antioxidants and OS biomarkers. When the plasmatic values of antioxidants are inferior to the normal or when blood ROS levels are much higher than healthy control, then antioxidant supplementation will be potentially positive, thence the importance of performing laboratory blood tests in order to determine the antioxidant status before starting intervention. Another interesting detail that may explain the beneficial effects in these trials was, probably, the short duration of treatment going from ten days to four months. Supplementation for a short period seems to be beneficial even when high-doses of antioxidants are used. Furthermore, short-term supplementation was likely to reduce chemotherapy-related toxicity and side-effects in cachectic patients, without affecting its anticancer potential [140].

### 6.4. Self-Prescription Supplements by Cancer Patients: An Alarming Phenomenon

**Figure 4:**
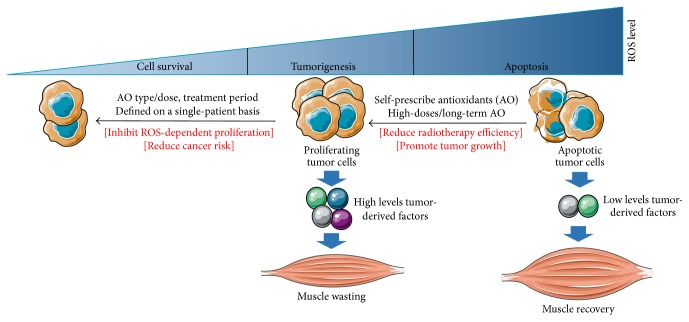
Hypothetical model for the eventual beneficial or deleterious interactions of antioxidants with tumor. ROS play a Janus-faced role by controlling both tumor growth and arrest. The levels of ROS produced within tumor depend on tumor type/localization and whether or not patient is undergoing radio/chemotherapy. Moderate-to-high ROS levels promote tumor proliferation, resulting in an increase in the levels of tumor-derived factors and the subsequent development of muscle atrophy. While high-to-excessive production of ROS activates tumor apoptosis and reduces the related catabolic response, the supplementation with antioxidants may decrease ROS at both systemic and muscular level but could also interact with tumor leading sometimes to undesirable consequences. For example, when excessive levels of ROS are produced within tumor, megadoses of antioxidants, used randomly, could increase tumor proliferation and/or inhibit apoptosis, by reducing oxidative damage in tumor cells. On the other hand, an appropriate use of antioxidants can decrease the risk of cancer development or even slow ROS-dependent cancer growth. The probability of reaping these antioxidant-related benefits could be much higher when supplementation is provided on a single-patient basis.

Patients are highly interested in vitamins and other antioxidant supplements, as they believe that these compounds are natural and beneficial for health [24]. The prevalence of supplements use is approximately 60% in lung, 49% in colon, and 35% in prostate cancer patients [141–143]. Previous studies have shown that the use of alternative medicine was associated with higher education, regular physical activity, fear of cancer recurrence, influence of family members, and participation in social groups [142, 144]. So,* how can this undifferentiated use of supplements by cancer patients impact tumor and anticancer treatment? *As mentioned in previous paragraphs, it seems that supplementation with antioxidants can reduce the efficiency of radiotherapy [24], but the limited number of results from clinical and preclinical studies prevented an evidence-based conclusion. Practitioners usually prohibit the use of supplements during chemotherapy or radiotherapy, as a preventive strategy against an unproven product that could be deleterious for patients' health [145]. Thus, a special attention must be given for cancer patients with comorbidity such as age-related eye disease, since they usually take antioxidant supplements as part of their treatment [24]. To maximize gain, patients not receiving or after achieving radio/chemotherapy, should be monitored for antioxidants use in the context of a well-defined treatment plan [146]. Supplementation with simpler antioxidant mixtures may be also preferred over complex cocktails [146]. Approximately, 50% of patients taking antioxidants or multivitamins did not inform their treating physician; the main reason was that physician did not ask about it [142]. Importantly, patients who discussed the use of supplements were less susceptible to using it [142]. Therefore, clinicians can better control the random use of such compounds by openly discussing with patients about their self-prescription of antioxidants and the potential harms of random use. As illustrated in Figure 4, we suppose that an autoprescription of megadoses antioxidants during a long period could protect tumor and reduce the efficacy of anticancer therapies.

### 6.5. Exercise: A Good Alternative to Antioxidants in Cancer Patients? 

Physical activity is well-known to produce moderate levels of ROS and induce hormetic adaptations within skeletal muscle [147]. Adapted activity promotes the expression of antioxidant genes (i.e.,* SOD1* and* GPX*) and increases GSH content, which in turn counteract muscular oxidative damage [147]. Additionally, adapted exercise evokes anti-inflammatory responses by producing high amounts of IL-4, IL-10, and IL-15 that antagonize the effects of proinflammatory cytokines and block the activation of the aforementioned procatabolic pathways [148]. We have previously reviewed the impact of physical activity levels on cancer progression and noticed that data available from the literature support a global positive effect of moderate exercise on tumor growth and survival in cancer patients [149]. In 2012, Battaglini and his team proposed their theoretical model of “Exercise Anticachectic Hypothetical (EACH) model.” They demonstrated that regular physical activity regimen can positively influence skeletal muscle myoplasticity, in leukemia and breast cancer patients [150, 151]. In other studies, the application of resistance or moderate endurance exercise program improved muscle function and decreased fatigue and proinflammatory cytokines production (i.e., IL-1ra and IL-6) in prostate and breast cancer patients undergoing radiotherapy [152, 153]. Globally, both resistance and endurance exercise improved muscle strength in early stage cancer patients [154]. However, there is a need for clinical trials to determine the effectiveness of exercise in cachectic patients with advanced stages of cancer [155]. It seems that moderate-to-high endurance exercise could be more suitable than resistance exercise to counteract muscle atrophy. In fact, resistance exercise results mainly in the activation of the anabolic Akt/mTOR pathway [156], but the anticachexia role of Akt is still a subject of debate and some experts in the filed consider the activation of Akt useless in the prevention of muscle wasting [157]. Additionally, recent evidence from animal studies suggest that moderate endurance exercise improves muscle mass [158], reduces fatigue, and extends survival, while resistance exercise worsens cachexia symptoms [157, 159]. Endurance is still the most used exercise mode given its capacity to drive metabolic adaptations in skeletal muscle, through activating mitochondrial biogenesis, improving the oxidative capacity of muscle, and increasing antioxidant activity and anti-inflammatory response [147, 160, 161]. Therefore, endurance exercise could be proposed in the early stage of disease for precachectic patients to delay the onset of cachexia and preserve muscle function. It is important to (1) individualize the level of physical activity based on the cardiopulmonary capacity and muscle strength of patient, (2) if possible, increase the intensity of exercise progressively to reap greater physiological adaptations, and (3) specify the treatment according to the primary end point of the study [162]. For example, a moderate-intensity endurance exercise could be proposed for patients to improve the cardiorespiratory function [163], while high-intensity endurance exercise could be prescribed to induce enzymatic adaptations in skeletal muscle [161]. The capacity of cancer patients with advanced stage of cachexia to perform exercise could be limited owing to anemia and cardiac dysfunction [157]. Thus, exercise could be replaced with other adapted activities such walking in order to avoid further muscle atrophy due to immobilization [164].

## 7. Conclusive Remarks and Future Directions

Emerging pieces of evidence suggest that the use of antioxidants cannot be standardized for all patients but should be individualized according to patient's need. The administration of high-doses antioxidants for a long period of time was most likely harmful in patients with gastrointestinal, head and neck, and lung cancer, especially if patients were smokers, undergoing radiotherapy, and/or with adequate antioxidant status, while, individuals with antioxidant insufficiency responded positively. In keeping with these findings, the small number of studies performed on cachectic cancer patients exhibiting low antioxidant status or high ROS blood levels indicated that a short-term supplementation (up to six months) was effective in improving physical function and quality of life. Interestingly, it seems that even in the same type of cancer an antioxidant treatment could be more or less advantageous depending on whether the patient is cachectic or not, hence the importance to add cachexia on the list of criteria used to select patients for an antioxidant intervention. In the light of these findings, random complementation cannot prevail. Patients may obtain antioxidants from fruits/vegetables (five portions of 80 g/day), while supplements must be reserved for those with particular needs. Accordingly, the measurement of blood antioxidant levels could be a simple approach to identify patients with specific deficiencies and, therefore, improve the use of such compounds in cancer cachexia. We might underscore that, given the multiorgan presence of OS in cancer cachexia, systemic antioxidant status does not necessarily reflect the redox events occurring in other organs like muscle, and the absence of antioxidant deficiency or high ROS rates in blood does not mean that muscles are spared from oxidative damage and atrophy. Nonetheless, this method remains more appropriate to the clinical context nowadays, where performing skeletal muscle biopsies is restricted for ethical and methodological reasons. Based on literature, natural polyphenols appear to be more effective than vitamins in cancer cachexia, probably, due to their capacity to modulate redox status, epigenetic pathways, and cellular senescence [165]. Furthermore, adapted physical activity could be a promising strategy for cachectic patients, as it positively affects muscle performance, OS parameters, and systemic inflammation. However, there is a real need for new clinical studies on a larger scale to further explore the role of antioxidants and physical activity in cancer cachexia. At the moment, a regimen combining moderate physical activity with an appropriate nutritional care could be the optimal way to improve quality of life, preserve muscle endurance, and naturally ameliorate enzymatic antioxidant defense in cancer cachectic patients.

## Figures and Tables

**Table 1:** Impact of allopurinol on cachexia symptoms in C26 mice. Balb/C mice subcutaneously inoculated with 1 × 10^6^ C26 cells have received daily dose of allopurinol (50 mg/kg/day) or vehicle (PBS). Mice weight was daily monitored and skeletal muscles were weighted at the end of the protocol. Fiber diameter was determined from at least 100–150 fibers per muscle histological section, stained with the Gomori method. Data are mean ± SEM (*n* = 8/group).

	Control	C26	C26-allo
Initial body weight (g)	23.6 ± 0.6	23.4 ± 0.6	23.9 ± 0.7
Final body weight (g)	25.9 ± 0.5	21.5 ± 2.1	20.1 ± 1.8
ΔBody weight (g)	2.3 ± 0.5	−2.2 ± 2.05^a^	−3.8 ± 1.3^a^
Soleus weight (mg)	7.1 ± 1.7	6.4 ± 2.3	7 ± 3.3
Gas weight (mg)	128.1 ± 14.4	94.5 ± 15.1^a^	91.5 ± 22.7^a^
EDL weight (mg)	10.7 ± 2.3	8 ± 1.8^a^	8.2 ± 1.5^a^
EDL fiber diameter (*μ*m)	41.62 ± 2.4	29.8 ± 5.7^a^	36.8 ± 5.2^b^

^a^ *P* < 0.001 versus control; ^b^ *P* < 0.01 versus C26; Gas: *Gastrocnemius*; and EDL: *extensor digitorum longus*.

**Table 2:** List of main clinical intervention studies with antioxidants on cachectic patients. One open nonrandomized trial (NRT) shows that, at the baseline, cachectic patients present higher ROS levels and lower GPx activity in blood samples comparing to healthy control subjects. Phases II and III studies show that a combination of antioxidants and other agents, including appetite stimulants (megestrol acetate, MA), anti-inflammatory COX-2 inhibitors (celecoxib, CXB), omega-3 rich fatty acid (eicosapentaenoic acid, EPA), enhancers of lipid *β*-oxidation (L-carnitine, L-CAR), and immune-modulatory agents (thalidomide, TMD), decreases the levels of ROS in the blood, augment the enzymatic antioxidant activity of GPx, and improve performance status (PS) in cancer cachectic patients. Data are presented in the table as mean values that reached statistical significant difference (*P* < 0.05). No statistically different values are replaced with NSD.

References	Cachectic patient population	Type of study	OS biomarkers (baseline) Cachectic versus healthy control		Treatment		Clinical outcomes (baseline versus treatment)		
			ROS (FORT U)	GPx (U/L)	AO types	Other agents	PS: ECOG score	ROS (FORT U)	GPx (U/L)
Maccio et al. 2012 [12]	Ovary, endometrium, and cervix cancer: 104	R-phase III, 4 mo	—	—	ALA and CS^a^	L-CAR + CXB + MA	1.75 versus 1.12	528 versus 444	6007 versus 7458

Madeddu et al. 2012 [13]	Including H&N, lung, and colorectal: 60	R-phase III, 4 mo	—	—	ALA, CS, PLP, and Vit A, C & E^b^	L-CAR + CXB + MA	1.7 versus 1.4	—	—

Mantovani et al. 2010 [14]	Including breast, pancreas, and colon: 332	R-phase III, 4 mo	—	—	ALA, CS, PLP, and Vit A, C & E^c^	MA + EPA + TMD + L-CAR	2 versus 1.5	NSD	NSD

Mantovani et al. 2006 [15]	Including breast, lung, and stomach: 39	NR-phase II, 4 mo	—	—	ALA, CS, PLP, and Vit A, C & E^c^	EPA + MA + CXB	—	468.5 versus 436.6	NSD

Mantovani et al. 2003 [16]	Including H&N, colon, and lung: 56	NRT, 10 d	403.4 versus 172	6770.6 versus 10813	ALA, CS, NAC, and Vit A, C & E^c^	—	Correlation with OS markers^*∗*^	403.45 versus 345.9	6770.6 versus 9263.7

^a^ *α*-Lipoic acid (ALA, 600 mg/day) and carbocysteine (CS: 2.7 g/day); ^b^N-acetylcysteine (NAC: 1800 mg/day), ALA (200 mg/day), CS (2.7 g/day), Vit A (30000 IU/day), Vit C (500 mg/day), and Vit E (70 mg/day); and ^c^polyphenols (PLP: 300–400 mg/day), ALA (300 mg/day), CS (2.7 g/day), Vit A (30000 IU/day), Vit C (500 mg/day), and Vit E (400 mg/day). ^*∗*^Cachectic patients with high ROS levels and low GPx activity exhibited poor performance status. H&N: head and neck cancer.
